# A Case Report of an Uncommon Association of Total Intracardiac Anomalous Pulmonary Venous Return and Tetralogy of Fallot

**DOI:** 10.7759/cureus.60493

**Published:** 2024-05-17

**Authors:** Hassnae Tkak, Aziza Elouali, Ayad Ghanam, Imane Kamaoui, Maria Rkain, Abdeladim Babakhouya

**Affiliations:** 1 Department of Pediatrics, Mohammed VI University Hospital, Faculty of Medicine and Pharmacy, Mohamed I University, Oujda, MAR; 2 Department of Radiology, Mohammed VI University Hospital, Faculty of Medicine and Pharmacy, Mohamed I University, Oujda, MAR

**Keywords:** children, echocardiography, venous return anomaly, cyanosis, congenital cardiopathy

## Abstract

Total anomalous pulmonary venous return (TAPVR) represents a group of anomalies consisting of a lack of connection between the pulmonary veins and the left atrium. All oxygenated pulmonary venous return flows directly or indirectly into the right atrium. Survival is only possible with a right-to-left atrial shunt. It remains rare, accounting for less than 1% of all congenital heart diseases. Its association with tetralogy of Fallot is much rarer and has been documented in medical literature as isolated cases. Early prenatal diagnosis, rapid surgical repair, and optimal postoperative resuscitation appear to be the best guarantee of a favorable outcome following total repair of a pulmonary venous connection anomaly.

Non-obstructed forms present as high-flow shunts with moderate cyanosis. The symptomatology of blocked forms is dominated by the obstruction to venous return; a clinical picture of respiratory distress with intense cyanosis and severe pulmonary arterial hypertension develops from the first days of life. Echocardiography is fundamental in diagnosing TAPVR. If the results are inconclusive, magnetic resonance imaging and computed tomography are appropriate alternatives for establishing a complete and accurate diagnosis.

We report a case of a two-month and 22-day-old infant who is a product of a twin pregnancy, presenting with a non-obstructed TAPVR associated with tetralogy of Fallot, and his twin who died on day 20 of life, likely due to a complex cyanotic congenital heart disease.

## Introduction

Total anomalous pulmonary venous return (TAPVR) and partial anomalous pulmonary venous return (PAPVR) are rare congenital heart malformations [1]. Isolated TAPVR is even rarer, occurring in approximately one case per 14000 to 17000 newborns, accounting for less than 1% of all cases of congenital heart disease [1]. In the normal state, the four pulmonary veins join together in the left atrium, whereas in the case of TAPVR, the pulmonary veins connect to the systemic venous system rather than to the left atrium [2,3]. The association between tetralogy of Fallot and TAPVR is rare and has been documented in medical literature as isolated cases [4].

Prenatal diagnosis can positively influence the prognosis, especially in cases of obstructed TAPVR, which typically require immediate surgical intervention after birth [1]. Despite ongoing advances in pediatric cardiac surgery, this malformation remains serious, necessitating individualized management [2].

## Case presentation

This is a male infant, two months and 22 days old, from a non-consanguineous couple and a well-monitored twin pregnancy carried to term by cesarean section. Prenatal ultrasound did not show any anomalies. The mother had gestational diabetes managed with insulin. He was born at 39 weeks of gestation with good adaptation to extra-uterine life. His birth weight was 2.9 kilograms. He was hospitalized on his second day of life in the neonatology unit for 10 days due to early maternal-fetal infection, along with his twin brother who passed away on day 20 of life due to respiratory distress and cyanosis refractory to oxygen.

In the interview, the mother reported episodes of cyanosis during exertion (such as crying or feeding) observed since birth. However, no signs of fatigue or sweating during feedings were reported. The baby was admitted with respiratory distress characterized by rapid breathing, cough, and nasal congestion, with a decrease in feeding frequency evolving one week before admission, in the absence of fever.

**Figure 1:**
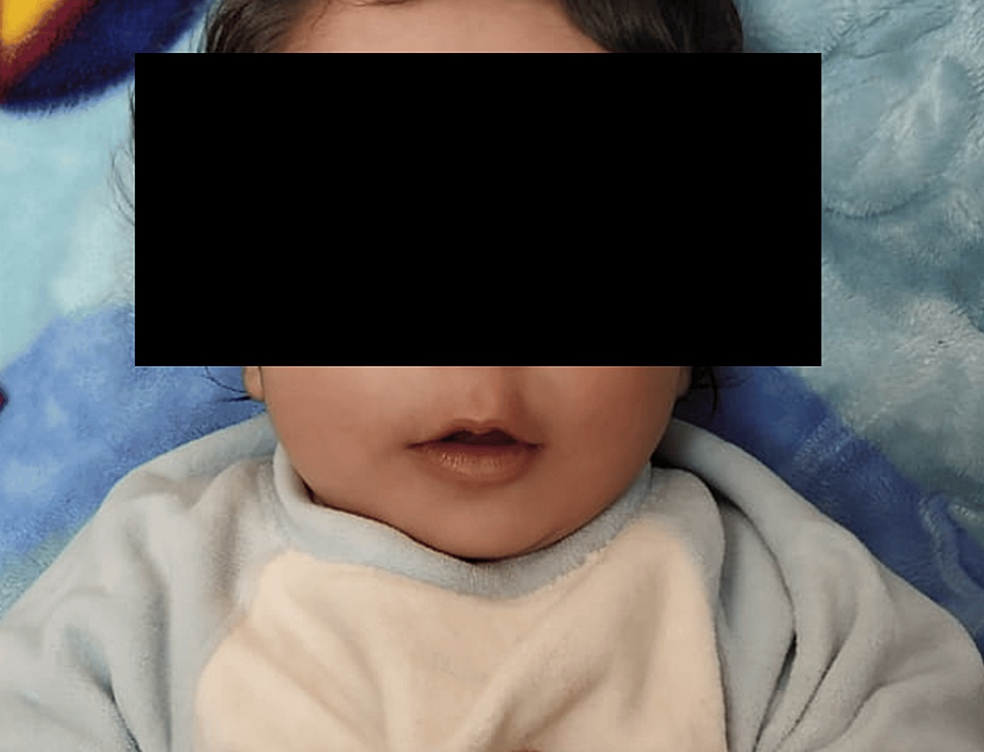
Moderate cyanosis of the lips

On admission, he was tonic, responsive, and smiling, with a normal weight of 4kg 900gms. He had refractory cyanosis on the hyperoxia test, with a saturation of 78% on room air and 84% on oxygen (Figure 1). He was polypneic at 42 cycles per minute and his heart rate was 135 breaths per minute. On pleuropulmonary examination, there was subcostal thoracic traction and a heart murmur, but no hepatomegaly.

**Figure 2:**
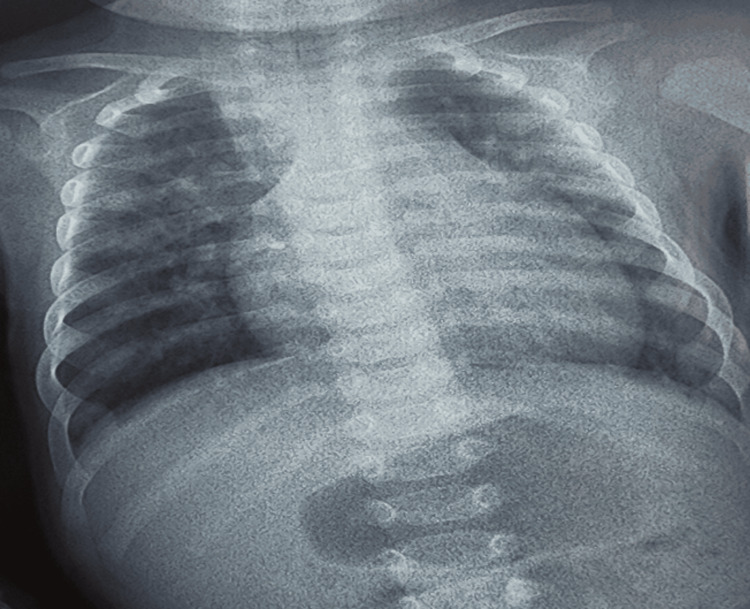
Chest X-ray showing cardiomegaly with pulmonary hypervascularisation

**Figure 3:**
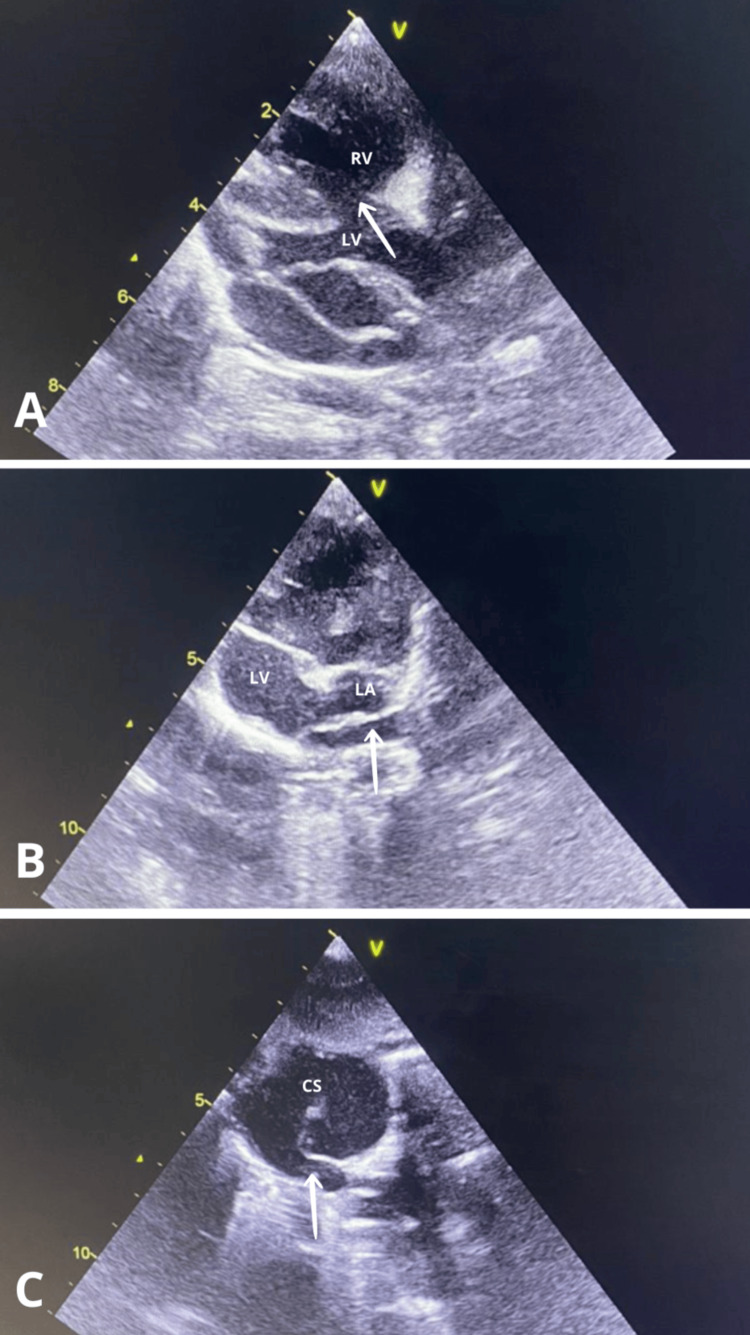
Echocardiography two-dimensional mode: (A) Ventricular septal defect (arrow); (B) The retroauricular collector where the pulmonary veins drain (arrow); (C) The collector of the pulmonary veins (arrow) which drains into the coronary sinus and then into the right atrium. LV: left ventricular; RV: right ventricular; LA: left atrium; CS: coronary sinus

The frontal chest X-ray showed cardiomegaly with a cardiothoracic ratio of 0.58 and increased pulmonary vascularity (Figure 2). Transthoracic echocardiography revealed a complex cardiac anomaly consistent with tetralogy of Fallot: Dextroposition of the aorta, right ventricular hypertrophy, ventricular septal defect, without pulmonary artery stenosis, associated with an unobstructed total intracardiac anomalous pulmonary venous return (PVR), type II: all four pulmonary veins drained into the right atrium via the coronary sinus (Figure 3).

**Figure 4:**
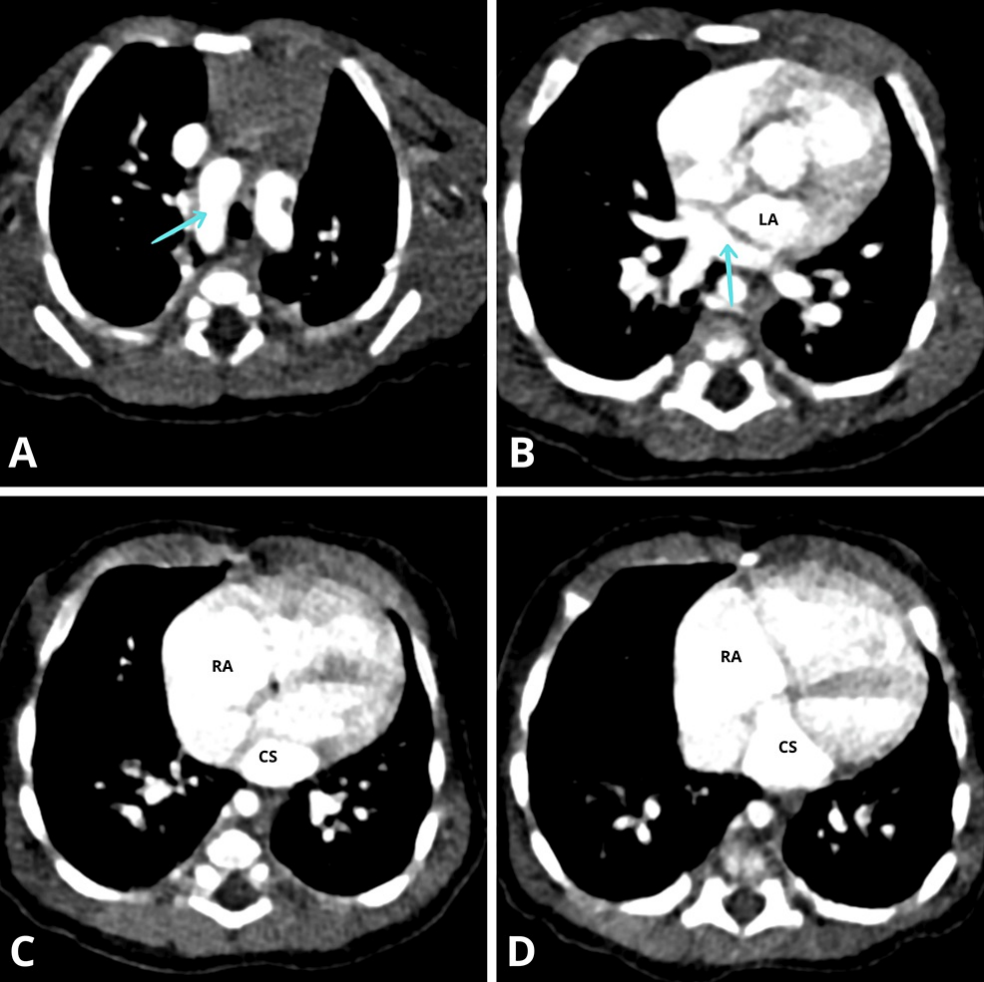
Thoracic axial CT angiography shows (A) dextroposition of the aorta (arrow); (B) retroauricular collector where pulmonary veins drain (arrow); (C) coronary sinus where the collector drains; (D) The coronary sinus draining into the right atrium. LA: left atrium; RA: right atrium; CS: coronary sinus

This was complemented by a thoracic angioscan, affirming the diagnosis of this uncommon combination of TAPVR and tetralogy of Fallot (Figure 4).

The infant was placed on symptomatic treatment for heart failure with good clinical progress, and then transferred to a specialized cardiovascular surgery center. The child underwent corrective surgery at the age of five months, and then died on postoperative day 3.

## Discussion

Anomalies of the PVR refer to a group of congenital heart malformations in which the veins draining oxygenated blood from the lungs to the heart exhibit connection abnormalities. Normally, these pulmonary veins join together to form a common pulmonary vein that connects to the left atrium of the heart. However, in PVR anomalies, the veins can connect abnormally to other parts of the heart, resulting in inadequate mixing of oxygenated and deoxygenated blood. Types of PVR anomalies include TAPVR, where all pulmonary veins connect abnormally, as well as PAPVR, where only certain pulmonary veins are affected. These anomalies can lead to cyanosis, respiratory problems, and other cardiovascular complications [3], with pulmonary vein stenosis being the most dreaded complication, often proving fatal [2].

The incidence of anomalous PVRs remains unknown, as partial forms, which represent approximately two-thirds of cases, are often asymptomatic. It is estimated to be around 1% to 2%. However, this incidence may be underestimated due to some cases that may go unnoticed, especially minor or asymptomatic forms in contrast to total forms, which represent <1% of all cases of congenital heart disease [1,3]. Advances in imaging techniques and neonatal screening programs can also influence the detection rate of APVR [1].

TAPVR is divided into four types according to the site of connection. In Type I, supracardiac, the most common form (40% to 50%), drainage from the collector occurs to the left towards a left vertical ascending vein, then to the innominate vein and the superior vena cava. To the right, drainage occurs towards a right ascending vein and the superior vena cava. In Type II, intracardiac (20% to 30%), drainage occurs to the right atrium directly or via the coronary sinus. Type III, infracardiac (10% to 30%), in this form the pulmonary veins typically empty into a descending vertical vessel through the hiatus, usually heading towards the portal system, directly or via the venous sinus, or towards the inferior vena cava or suprahepatic veins. In mixed forms, drainage can occur above and below the diaphragm [2,3]. In Type III, it is common to encounter an obstruction to venous return, whether in the vertical path between the aorta and the left atrium, during passage through the diaphragm, through the hepatic sinusoids, or due to closure of the venous sinus [3].

In TAPVR, there is a right-to-left intracardiac shunt where oxygenated and deoxygenated blood mix completely in the right heart chambers. Overloading of the right heart chambers occurs due to the abnormal influx of pulmonary blood into the right venous system. This leads to dilation of the right atrium, right ventricle, and pulmonary artery, while the left chambers retain their normal size or decrease. Partially deoxygenated blood is then redistributed into the systemic circulation via an atrial septal defect (ASD) or a persistent foramen ovale, resulting in cyanosis, particularly notable in Type III cases. In the presence of venous return obstruction, especially in Type III cases, significant pulmonary vascular overload occurs, accompanied by edema and pulmonary arterial hypertension, necessitating urgent diagnosis and surgical treatment [3].

The presence of associations with other malformations is possible: an ASD or a persistent foramen ovale is always observed, without which the malformation would be incompatible with life. Other malformation associations may also be present, such as a single ventricle, an atrioventricular canal, tetralogy of Fallot, as observed in our patient, an anomaly of the systemic venous system, a left triatrial heart, asplenia syndrome (Type III), and biliary atresia [2,3].

The association between tetralogy of Fallot and TAPVR is rare. Its incidence has been reported at 0.25% (3 out of 1,183) [5] and 0.34% (2 out of 592) [6] among all patients with tetralogy of Fallot. In some studies, the diagnosis of tetralogy of Fallot associated with total anomalous pulmonary venous drainage was established either at autopsy of patients who had undergone incorrect surgical intervention or intraoperatively [4]. Although some studies have highlighted difficulties in identifying these lesions by echocardiography [7,8], the presence of a dilated coronary sinus with improper visualization of the pulmonary veins should raise suspicion of associated cardiac type of total anomalous pulmonary venous drainage. In the past, the diagnosis was often suspected by echocardiography but was confirmed only at cardiac catheterization. In most cases of tetralogy of Fallot associated with total anomalous pulmonary venous drainage, the initial symptoms appear to be those of tetralogy of Fallot. The coexistence of these two malformations leads to a chronic overload in volume and pressure on the right side of the heart, resulting in dilation of the right atrium and ventricle, as well as underfilling of the left atrium and ventricular [4].

The diagnosis of TAPVR requires comprehensive clinical evaluation as well as additional tests. Clinical manifestations of TAPVR may vary depending on the patient's age, the severity of the anomaly, and the presence of other associated cardiac malformations. However, some indicative signs may be observed in patients with TAPVR, such as cyanosis, heart failure, and increased pulmonary vascularity [3,9]. In cases of severe pulmonary venous obstruction, especially observed in newborns, particularly Type III, patients typically present marked respiratory distress and severe cyanosis. Without prompt corrective surgical intervention, the patient's prognosis becomes critical. Significant left-to-right shunting can lead to cardiac failure, manifested by symptoms such as tachycardia, hepatomegaly, and tachypnea. This failure may progress gradually during the first month of life, following the decrease in postnatal pulmonary resistances, which explains the presence of asymptomatic neonatal forms. Surgical intervention is essential to ensure the patient's survival [10].

The diagnosis of TAPVR is crucial for determining appropriate management. Imaging is pivotal during this diagnostic journey. Chest X-ray can provide useful additional information for diagnosis. Although not specific to TAPVR diagnosis, it may show certain indirect signs suggesting the presence of this cardiac anomaly. These signs typically include cardiomegaly, resulting from increased blood flow to the heart, as well as increased pulmonary vascularity due to abnormal mixing of oxygenated and deoxygenated blood. Echocardiography, in particular, is a commonly used imaging modality to identify TAPVR characteristics. It can reveal several anomalies, including the absence of pulmonary vein drainage into the left atrium, the presence of the retro auricular collector with a stenosed drainage vein, and the presence of ASDs or persistent foramen ovale. Additionally, it can highlight dilation of the right heart chambers and pulmonary arteries in certain types of TAPVR (Types I and II). Visualization of a supra- and sub-diaphragmatic vein with the flow away from the heart is pathognomonic of Type III total anomalous return. Echocardiography also allows the assessment of pulmonary pressures, although it is generally not sufficient for precise mapping of the abnormal venous return [3,9].

When echocardiography does not provide the necessary data, MRI and CT are the methods of choice for visualizing congenital anomalies of the pulmonary veins to establish a comprehensive and accurate diagnosis of TAPVR and for a more detailed evaluation of cardiac anatomy. In most cases, MRI provides precise anatomical and functional data without using ionizing radiation. CT offers higher spatial resolution and shorter imaging times, but at the expense of exposure to ionizing radiation. The speed of CT is useful in newborns and severely ill infants with TAPVR [11].

Prenatal diagnosis of TAPVR, in particular, remains challenging despite technological advancements in fetal ultrasound and echocardiography, with prenatal detection rates of isolated TAPVR still reported as low as 2% to 10% in some studies [12,13]. However, it contributes to improving outcomes, especially in cases of obstructed TAPVR, which typically require urgent surgical intervention after birth [1]. A large multicenter series of 424 cases of TAPVR conducted by Seale et al. also reported low diagnostic rates during screening, with only 1.9% of cases diagnosed by prenatal echocardiography but none during routine screening [1,12]. Laux et al. reported a higher success rate with routine screening, detecting six out of 10 cases prenatally diagnosed. Therefore, the success of diagnosis during routine screening varies, but according to the literature, we continue to miss 40% to 100% of cases [13].

In TAPVR, the connection between pulmonary return and the cardiac chambers is established by a collector that returns blood to the right chambers. The urgency of surgical treatment is linked to the obstruction of this collector. Different techniques have been developed to treat the various forms of abnormal PVRs, which are subdivided into supra-, intra-, or infracardiac depending on the site of connection of the collector. Long-term outcomes depend on whether there is involvement of the pulmonary veins themselves, with secondary stenosis of the pulmonary veins remaining a severe complication often with a reserved prognosis. Cases associated with other malformations require a multidisciplinary approach, often involving surgical intervention to correct cardiac anomalies, and long-term regular follow-up. Overall prognosis has greatly improved over the years, and the surgical outcomes of conventional techniques from recent decades are satisfactory [2]. Without surgical intervention, the mortality rate reaches 80% within the first year [14].

## Conclusions

TAPVR are congenital heart malformation characterized by abnormal connections of the pulmonary veins to the heart. Their diagnosis relies on imaging examinations such as echocardiography, MRI, and CT scans. Treatment often involves urgent surgical intervention, especially in cases of severe pulmonary vein obstruction. Prognosis has improved with advancements in surgical and diagnostic techniques, including prenatal ultrasound and echocardiography.
